# Extracts

**Published:** 1882-09

**Authors:** 


### Extracts.

CEMETERIES.—The dangers to the public health to which places of burial may give rise are of two kinds—viz., the contamination 1st of *air* by the gaseous and volatile, and 2nd, of *drinking water* by the liquid and soluble, products of decomposition.

1. *Contamination of Air*—This may take place in several modes. The gases evolved from putrefying bodies may make their way to the surface through pores or fissures in the ground, or may pass into open graves dug in their neighborhood. Or they may diffuse themselves laterally through the ground air and be drawn up into the interior of houses. Or noxious emanations may be given off from putrid drainage water, whether baled out of graves and thrown upon the surface, or draining into open channels or water courses. Thus nuisance and danger to health may be occasioned, not only to grave diggers and persons attending funerals, but also to the inhabitants of houses in the neighborhood of the burial ground. To obviate these risks it is necessary that the number of decomposing bodies in a given portion of ground should not at any time be so great that the gaseous products cannot be oxidized into harmless substances in the interstices of the soil, or taken up by vegetation; that a sufficient depth of earth intervene between corpses and the surface; and that the soil be of a suitable nature and properly drained, the drainage water being innoculously disposed of. Furthermore, since the atmospheric contamination which has to be especially guarded against is that of the air in the interior and neighborhood of human habitations and frequented places, it is necessary that the place of burial should be in an open situation, and at a sufficient distance from dwellings, in order that any effluvia arising from it may be diluted by diffusion, or dispersed by the winds, so as not to find their way in an injurious state of concentration to places where they will be liable to be inhaled.

2. *Pollution of Water.*—Foul liquids from graves may enter and pollute a stream, or wells in the vicinity of a graveyard may be injured by percolation from it, and in either case, if the water be used for drinking, injury to health may be occasioned. The liability of wells to pollution obviously depends partly upon their proximity to it and partly upon the configuration and geological structure of the ground. Thus, an intervening impervious bed of clay will prevent foul matters from reaching a well, and filtration through a sufficient distance of porous ærated soil decomposes such matters into harmless inorganic substances, which are fixed by the soil, or taken up by plants. It is necessary, therefore, in order to obviate risk from this cause, that a cemetery should have a suitable soil and be properly drained, and that it should be at a sufficient distance from subterranean sources of water supply, and in such a position with respect to them that the percolation of foul matters from one to the other may be impossible.—*Ext. from Tenth An. Report of Local Government Board, in Sanitary Journal, Glasgow.*

---

GUNSHOT WOUND OF ABDOMEN TREATED BY OPENING CAVITY AND SUTURING INTESTINES.—Amos Ray, colored male adult, wound received about 10 a. m., from discharge of pistol (32 calibre, Colt's) in hand of a fellow laborer with whom there had occurred a difficulty. The party firing was in front of Ray at the time and only distant some five paces. Patient had been brought to hospital a distance of several miles.

The orifice of entrance of ball was found midway between the umbilicus and pubes, a little to the left of the mesian line. There was general abdominal pain, and an inability to pass water, with symptoms of slight shock. The bladder was emptied by a catheter, and the urine found of natural character; a little blood escaped from urethra, which was sensitive and slightly strictured at two points.

At 8:30 p. m., abdomen was slightly swollen and tender, and in addition there was complaint of pain at end of spinal column just above the anus. The oiled finger passed into the rectum discovered an orifice through the posterior wall of the gut, and this corresponded with a depression in the bony tissue of extreme end of the sacrum. Dr. Kinloch determined upon an operation as offering the best chance for life under the very gloomy prognosis. At 9 p. m. the necessary preparations having been completed patient was chloroformed and the operation performed under carbolic spray and with full antiseptic precaution. The cavity was opened by an incision in the median line from the umbilicus nearly to the pubes. The intestines were turned out, and carefully examined.

There were discovered five perforations in the calibre of the small gut, and two in the mesentery. The wound in the rectum, after very careful examination, could not be discovered. The edges of all the intestinal wounds were trimmed with the scissors, and the wounds closed with fine carbolized silk sutures.

There was considerable blood in the cavity and but very little fecal matter. The cavity was carefully sponged out, and cleansed with a warm two per cent. carbolized solution. The abdominal wound was closed with silver sutures except at the lower angle where a rubber drainage tube was inserted. Carbolized oiled lint, several layers of borated absorbent cotton, and a broad bandage completed the dressing.

Patient was ordered gr. 1 of opium every four hours. Hot bottles to extremities. Internally nothing more than pellets of ice *ad libitum.*

The patient died in the afternoon of the next day.

Autopsy revealed the fact that the mesentery and omentum had five perforations, and the intestines four, two or three were orifices of entrance and two of exit. All the openings but one were closed, this, apparently, had escaped detection. The ball had passed downwards and slightly to the right, entering the rectum just below the recto-vesical fold, and traversing the sacro-coccygeal articulation, scaling off a piece of the sacrum and imbedding itself in the glutei muscles from whence it was extracted. There was a very little fecal matter in the abdominal cavity together with some bloody serum. The whole mass of the small intestines exhibited signs of acute inflammation, and here and there presented ecchymotic patches.

While this adds another fatal case to the list of those in which similar treatment has been instituted, it does not alter my belief in the correctness of the practice in the class of injuries presenting so gloomy a prognosis. Though I cannot feel as hopeful in regard to the future of such practice, as my distinguished friend, Dr. Sims, nor partake of that degree of enthusiasm which comes of the success in ovariotomy, I do look forward to better results than have been so far obtained. There is, strictly, no ground for comparing ovariotomy with the operations for intestinal lesions unless it be in contrasting the conditions in the two proceedings to determine at once how much more serious must ever be the prognosis in the latter. Listerism, however, and much of knowledge acquired by ovariotomists will embolden many surgeons, who up to this time have favored the passive policy in the treatment of intestinal wounds. We shall soon have evidence of this and fuller statistics upon which to base conclusions.

From the report above, it will be seen that the autopsy disclosed the fact that one of the perforations in the intestine was overlooked at the time of operation. This has occurred before in at least one reported case, and such an accident is a strong argument with many who are opposed to the active treatment. This failure to close one of the wounds would, with most surgeons, be regarded as a fatal mistake—one that would always seal the fate of the patient.

I think this, by no means, a legitimate conclusion. Where drainage can be insured, and the cavity washed out, such a calamity, fearful though it be, may at times be successfully prevented.

My failure to find the wound in the rectum, which I knew to exist, was a sad disappointment to me, but I looked for help to the drainage tube. But for this failure I should have closed the cavity.

I do not think that open wound of the intestine determined the fatal result. The autopsy proved that there had been but little escape of intestinal matter into the cavity, or, that, such discharges had been washed out, by the injections thrown into the cavity.

Death, apparently, was neither from peritonitis nor septicæmia. The little suffering of the patient after the operation was a marked feature. The opening of the abdominal cavity seemed to save from suffering, if it did not save life. The lateness of the operation, (eleven hours after injury) militated against its success, and the numerous perforations of the gut, perhaps rendered success impossible.—*R. A. Kinloch, M.D, in North Carolina Medical Journal.*

---

New York Pathological Society.—*Membranous Dysmenorrhœa.*—Dr. Newman: Patient æt. 26, married three years, no children. Came under treatment three years ago. Three years ago she was thrown down and kicked in the left side. In April last again came for treatment for membranous dysmenorrhœa. A distinct tumor was found, which was entirely disconnected from the uterus. In August, after severe pain in left side she felt something give way, and a copious discharge of blood from the uterus followed. She was relieved until October, 1881, when she was troubled with flooding and expulsion of membrane at her monthly periods, which were, however, regular. She was treated by dilatation of the uterus, application of carbolic acid and tincture of iodine. She was then well until March, 1882, when she passed such large pieces of membrane that some physicians told her she was pregnant and wanted to confine her for child. The masses expelled were found to be exfoliated intestine, which had been passed per rectum. The interest of the case was in the diagnosis.

*Perityphlitis from Cherry Stone in Appendix Vermiformis.*—Dr. Ferguson: May 1st, 1881, the patient awoke with violent pain, right iliac region. There was no history of traumatism. On the ninth day of his illness, which had been chiefly characterized by persistent vomiting and pain, he came into the New York Hospital. Respirations shallow, pulse feeble, temperature 102, face anxious. On examination a tumor was found 6x4 inches, exquisitely tender. It was elicited that the day before his illness he had eaten cherries and swallowed the stones. He died twenty four hours after admission.

On post mortem pus was found in the peritoneal cavity, and a large sac in the wall of the ileum, and appendix filled with pus.

*Intestinal Intussusception.*—Dr. J. Lewis Smith: Negro girl æt. 7, health good until five months ago, then complained of colicky abdominal pains which soon became more frequent and severe. Dr. Smith saw her March 13th; the pulse was normal, there was no marked febrile movement In intervals of pain she was apparently in good health. Evacuations one or two daily, were of a diarrhœal character. There was no evidence of a tumor, though the patient was examined carefully for intussusception. There were fugitive pains in other parts of the body. Intestinal catarrh and neuralgic pains were diagnosed. She gradually lost strength and died from exhaustion. There had been at no time marked vomiting. On opening the abdomen attention was drawn to the appearance of the small intestine, which was markedly congested as a result of enteritis. The ascending and transverse colon were not in view at all. In intussusception the starting point is prolapse of the ileum through ilio-cæcal valve or inversion of the caput coli. This commenced in the former way. The displacement must have existed from 3 to 5 months. If the diagnosis had been made the patient might have been relieved. Dr. Smith had found that if intussusception occurred after the age of two-and-a-half years there would be found some antecedent disease of the intestine. In answer to an inquiry of Dr. White, he replied that he regarded water better than air for the expansion of the intestines in these cases.—*Med. Gazette.*

---

Caustic Potash Treatment of Carbuncles—Prof. Post is enthusiastic over the use of stellate and crucial incisions in the treatment of carbuncles; he states that he has had no personal experience in the use of caustic potash.

I am just as enthusiastic about the potash treatment; and from quite an extended experience with it I can confidently recommend it to the profession, using, however, certain precautions in the application, which I have gradually learned.

I will give the outline of the treatment of one carbuncle. He came to me with one about the size of a silver dollar, it having been considerably irritated from its position (under the waistband). The application was made in the manner above described, boring deeply until I judged I was about half way into the substance of the carbuncle, meanwhile protecting the sound skin by swabbing with acetic acid. In spite of this the eschar at first, although only as large as the big end of a lead pencil, became as large as a silver half dime. The caustic action, when carried far enough, was stopped by the use of the acid. In a little while all pain had ceased, and the wound was left with a simple dressing of absorbent cotton. The result of the application was the formation of a large, dry, black and firmly adherent scab; but what was most remarkable, besides the absolute relief to pain, the indurated and brawny mass was reduced to less than half its former size. In a few days the eschar was removed by a few poultices applied at night; the induration was then gone, leaving a simple ulcer which rapidly healed under iodoform.

I have never tried the insertion of peas of caustic potash, but I would unhesitatingly condemn it as unscientific. In this manner, in a thin-walled abdomen, perforation with fatal result is among the possibilities.

In conclusion, then, I would first call attention to the method—continuous and immediate action of the caustic potash until the desired result is attained and then neutralizing the alkali with C. P. acetic acid on the skin, or dilute acetic acid if on a mucous membrane, leaving the patient only when the caustic action has completely stopped; secondly to the advantages—relief of pain, its bloodlessness, the perfect control of the operator over the extent of the caustic action, the simple after treatment, and the rapid recovery.—*B. F. Leonard, M.D., in Maryland Med. Monthly.*

---

DIAGNOSIS OF HIP-JOINT DISEASE.—Hip-joint disease is liable to be confounded with a good many affections.

In the first place, you may mistake it for a congenital dislocation. To settle this point, measure the trochanter major down to the internal malleolus and see if it corresponds with the other side. It may be mistaken for acute articular rheumatism, neuralgia, sacro-iliac disease, hysteria, worms, teething, pain referable to destruction of the anterior crural or obturator nerve. You put the child on its back, press upon the popliteal space and up comes the vertebra, showing Pott's disease or vertebral caries. Psoas abscess is another affection, which is confounded with hip joint disease; also inflammation of the bursæ about the hip-joint.

The diagnosis must be made by careful examination and from the history of the case and symptoms.

If you have disease of the sacro-iliac synchondrosis, you will in the first place have absence of local symptoms. There will be no pain or tenderness over the hip. You will have no effusion, but you may have rigidity, especially if the nerves of the abdomen are irritated, but on the other hand in hip joint disease you have pain and tenderness on pressure, pain on pressing the surfaces together. Then you have the history of the case, the difference in deformity, etc. Suppose it be a case of hysteria, you may determine this by ether. The moment the patient begins to come out from under ether with hip-joint disease, and you move the joint, muscles will contract again. On the other hand, before perfect consciousness has occurred, she will move the joint and then contract the muscles again on recovering consciousness The one contracts in semi-consciousness and the other does not. If it be the psoas abscess, you have the absence of local symptoms, but you have pain oftentimes of the knee because the obturator and anterior crural nerves are involved. Chronic rheumatic arthritis may be mistaken, so that you must examine minutely into the character of the pains, their action and the causes that give rise to them.—*Joseph D. Bryant, M.D., in Med. Gazette.*

---

NOCTURNAL FRIGHTS IN CHILDREN.—"Sudden and transient outbursts of temper and strangeness in children should always make one search most carefully for signs of epileptic proxysms, which may be only slight—transeint pallor and vertigo—we should inquire for nocturnal incontinence of urine." These words of Dr. Jackson had a striking illustration in the case of a little, boy æt. seven years, with an epileptic history in his family. He had been liable for six months to awake in the night in a very disturbed state, and next morning would say it was his head; he could be roused completely, and then fall asleep. One night he went to bed in usual health, and whilst asleep was taken with a seizure described as a "great fright;" he kept crying out for half an hour, and would answer questions without being properly awake; he complained of his head, and desired his mother to go down stairs, although it was midnight. Next morning, whilst cutting out some pictures, he fell on the fender in a complete epileptic fit. He was brought to the hospital in a semi-comatose state, with a temperature of 102.4, which did not fall to normal for forty-eight hours.

An equally clear illustration is as follows: A girl, æt. seven years, with epilepsy in her family, was frightened by having been told that there was a black man in her room. She went to bed as usual, though her mother thought she looked "wild about the eyes." In an hour she woke up screaming, leaped out of bed, threw up the chamber window, and sprang out. Fortunately, her mother was in the yard, and caught her. She appeared alarmed, and screamed fearfully; she continued terrified during the night, and was languid for several days. Since this occurrence, she had been subject to fits of sudden violent screaming, without warning, lasting for ten minutes, and followed by heavy sleep. She was not coherent in the fits, but seemed to be greatly alarmed.—*James Russell, M.D., in Birmingham Medical Review.*

---

Antiseptic Treatment of Wounds.—I have in the foregoing remarks simply endeavored to draw your attention to the fact, that antiseptic surgery can be represented *solely*, neither by Mr. Lister's nor by any other method, but that the treatment of wounds must be based upon a firmer, more fully established basis than that which the germ theory affords. I have striven to demonstrate the fact that all good surgery is antiseptic; that surgery, in order to be good, must be directed to the prevention or to the control of inflammation, which is the "fons et origo" of all wound accidents, and that therefore, the method of wound treatment which most perfectly fulfills these indications may most deservedly claim to be antiseptic. The points I have endeavored to sustain in this paper may be conveniently formulated into the following propositions:

1st. The germ theory of wound infection is not established.

2nd. The antiseptie treatment of wounds after operations and injuries, is not limited to Listerism nor to any other special method, but is based upon broad, general principles.

3d. Antiseptic surgery embraces every condition or agent that tends to prevent putrefactive changes in wounds, or to remove or neutralize the effects of such changes when they have occurred.

4th. All wounds are healed by reparative inflammation.

5th. All wound accidents are the result, either directly or indircctly, of destructive inflammation.

6th. The antiseptic treatment of wounds, properly considered, consists first, of such means as will restrain inflammatory action within reparative bounds; and second, of such means as will subdue excessive action and remove or neutralize the effects of destructive inflammation.—*W. T. Briggs, M.D., in Nashville Jour. of Med. and Surg.*

---

Boiled Milk in the Summer Complaint of Children. —I was very much surprised to notice in a late number of a leading medical journal an editorial in which boiled milk is recommended as an article of diet in the summer diarrhœa of children. I know it has from time immemorial been the custom among mothers, grandmothers and nurses to stuff babies with boiled milk as a curative measure in this complaint, and that not a few physicians were following their example, but I had no idea that this treatment had been accepted by the profession, although I do not now remember to have seen any objection raised against it.

From about the first of July to the last of August, annually, I am obliged to repeat daily, and some days very frequently, my objections to this practice, and in some cases its mere stoppage was sufficient to effect a cure.

Milk contains albumen; boiling coagulates that albumen: What is harder to digest than coagulated albumen? The quality of pepsin is tested by the amount of coagulated albumen a given quantity will dissolve. You might as well feed your babes on cheese and hard-boiled eggs; these articles will, like boiled milk, check for a time a diarrhœa, by reason of their difficult digestion, but that very difficulty of digestion will, sooner or later, produce a diarrhœa or increase one already existing. Give the babes milk and plenty of it, but do not boil it.—*H. V. Swearingen, M.D., in Cincinnati Lancet and Clinic.*

---

Rectal Alimentation.—Dr. W. Joseph Tyson, F. R. C. S., Folkestone, in a paper on this subject in *British Medical Journal* says:

1. *Anatomically*, the rectum is not ill-suited for the purpose of feeding. It is extremely well supplied with blood vessels, which have a most free anastomosis; in fact, its mucous membrane is thicker and more vascular than that of any other part of the large intestine.

2. With respect to *absorption*, it has been said that substances known as colloids, such as albumen, gelatine, starch, etc., can not pass into the system until they are converted into crystalloids; and, as this change has been supposed not to take place in the rectum, the giving of enemata containing colloids, such as beef tea, eggs, etc., has little or no nourishing effect upon the body; and the brandy which is frequently added, only tends more to increase the colloid properties of the aforementioned foods, and therefore to render still more nugatory the use of these enemata. Graham supposes the coats of the stomach to dialyze the food during digestion, absorbing the crystalloids and rejecting the colloids—an action favored by the thick coating of mucus which generally lines the stomach; but Miller, after having quoted the above paragraph, goes on to say that "this suggestion probably requires some limitation—otherwise starch, gelatine, and other colloids, unless previously converted into crystalloids, would be wholly unabsorbed after they had been swallowed." The starch, as far as the stomach is concerned, is converted into a crystalloid by the saliva; and the starch which escapes being made dialyzable in the mouth is made so in the duodenum by the action of the pancreatic juice. This is the reason, probably, why sugar has been recommended to be added to nutrient enemata. Whether the rectum has the power of changing colloids into crystalloids, is perhaps doubtful; but results which have and do now follow the use of alimentation by the bowel, are too evident to leave any doubt that the rectum possesses properties by which nutrient injections, if not wholly absorbed, are certainly partially so.

3. *The operation of administering an enema* requires to be carefully and skillfully done. Anyone who has given these injections by means of the ordinary ball syringe, must have felt the inconvenience of this, the usual mode of procedure. If the ball be not quite full, air will probably be injected into the rectum, to the annoyance of the patient; and, even when the ball is full, great care must be exercised not to spill any of its contents on the bed. The best mode is to take a piece of india rubber tubing, two or three feet long. At one extremity fix a small piece of bone, resembling that which is attached to an ordinary Higginson's syringe; to the other end of the tubing attach a funnel. When the injection is to be used, the patient is placed on his side, the bone extremity of the apparatus oiled, and placed into the bowel, the other end raised, and the prepared enema is now poured into the funnel, and runs easily and comfortably into the rectum; the rate of progress can be increased or diminished according as the funnel is raised or lowered, or the food can be arrested at any time altogether by just nipping the tube below the funnel by the fingers of the hand holding it.

4. *In what cases should recourse be had to rectal feeding?* I would recommend it in all cases where obstinate and constant vomiting has existed for four days, or even before;of course, if the cause be a removable one this should be attended to at once. Then there are a large number of cases in which rectal alimentation might be used beneficially as a means of treatment or even cure; such as painful diseases of the stomach, including gastric ulcer, cancer, dilation, or, again, in some affections of the bowels.

5. *Composition of nutrient enemata.* Hot water can scarcely be regarded as a food; yet, in some cases of collapse, the injection of it, about the temperature of the blood, might very reasonably be given. In many conditions of partial stoppage of the circulation, an addition to the volume of the blood has been successful, at least temporarily, in re-establishing the action of the heart.

Nutrient enemata have been in the past, and are often now, made with beef tea, milk, the yolk of an egg, and a little brandy, either separately or combined; in bulk not exceeding three ounces, and given every two, four, or six hours, according to the exigencies of the case. Although the above have done good, their value has been enhanced since the introduction of artificial digestives. Pepsin in its various forms, and hydrochloric acid, were long used in stomach digestion, before their value was recognized in rectal alimentation. Pepsin is now very much replaced by the preparations of pancreatine, the latter possessing the double power of acting on proteids as well as on starch. The two ferments which have the property of changing the proteids into peptones, and the starch into sugar, are called respectively proteolytic and diastic; and, on account of this double property of pancreas, the preparations of the latter have come very much into vogue.

Enemata of blood have been recommended, and in some cases successfully tried. Three or four ounces of defibrinated blood having been injected into the rectum at night, no trace was found in the evacuations the next morning. Ox's blood has generally been employed. It must be fresh and defibrinated before use, and two or three ounces may be injected every two or three hours; but if there be any stomach-digestion going on, it may be less frequently used. In order that there may be no delay in its use, it can be obtained already prepared, concentrated and preserved in tins. To prepare the injection, the concentrated blood is dropped into the warm fluid to make the enema, a fluid drachm representing a fluid ounce of ordinary blood.

The success so far attending this novel mode of treatment is certainly sufficient to encourage an extended trial. —*American Practitioner.*

---

Tea and Coffee as Nervines.—Dr. Lewis Shapter, in *The British Medical Journal*, writes as follows on the value of tea and coffee as nervines:

The only restoratives to the nervous system which have stood the test of time, popular acceptation, and practical usefulness are infusions of tea and coffee, and such like beverages. It needs no medical knowledge to speak of the increased sense of respiratory power and muscular vigor; the promotion of general comfort and warmth; the cooling of the body, the result of the slight increase of respiratory functions; and the general restorative feeling when the body is overcome with fatigue, which is so generally and justly attributed to the use of tea, or, as will be presently shown, its active principle, theine, in the daily experience of life.

Failing, however, popular acknowledgment, medical and scientific research point to one and the same conclusion, that infusions of tea and coffee are restoratives to the nervous system, and indirectly act as sustainers to the circulation. Chemistry has shown that this action is dependent not upon the multiple constituents of the infusion of the leaf and berry, not upon any complex association of materials, but simply and entirely upon the presence of one substance—theine—which is the active and essential principle of all these and allied substances, and is known to us in the form of silky prismatic crystals, soluble in water. The quality of tea or coffee is, therefore, properly determined by the percentage of theine it contains, which varies from three to six per cent., and not by the delicacy and fullness of flavor which is imparted by the volatile oil. Medically speaking, this theine has a totally distinctive action from the infusions of which it forms a part. In the form of an infusion of tea or coffee, we have to deal with a large proportion of astringent matter, in the form of tannic acid, and with the presence of the essential oil, which is an excitant to the nervous system, and is the substance to which must be ascribed disorders of the nervous system which result from tea-drinking, such as palpitation of the heart and sleeplessness. The theine, upon the other hand, of which there is about one-tenth of a grain in an ordinary cup of tea, is the restorative agent to the nervous system, and is opposed, in its therapeutic properties, to the action of the essential oil. The infusion, therefore, of tea, may induce palpitation in a heart liable to excessive or incoordinate action; but theine, on the contrary, may be looked to, therapeutically, to quiet palpitation. The infusion, by being an excitant, may prevent sleep. Theine, by being a restorative and an indirect sustainer and regulator of the circnlation, may induce sleep. Individual medical investigations have, more than this, attempted, from time to time, to show that the action of theine is allied to that of quinine, as, in medicinal doses, the one has proven curative in allied diseases as well as the other; and moreover, that the nutritive value of theine may bear comparison with that of kreatine, one of the principles of meat.

All this is sufficient to show that theine is already a tried and proved agent, with a recognized dietetic position; and, if it has not hitherto occupied a place supplanting the use of alcohol, it is because (1) it has remained unrecognized as the active and essential principle upon which all the good qualities of tea and coffee, as restorative beverages, really depend; and (2) it has not been presented to the public in a form which would tend to render it acceptable or agreeable. In the form of an aerated water, theine offers the means of supplanting the abuse of alcohol. It forms an agreeable beverage, imparting a somewhat dry flavor to a distilled water, which may be sweetened and flavored to taste, and it offers a means for awakening scientific thought to the study of a simple remedy for a national abuse.—*Boston Journal of Chemistry.*

---

TREATMENT OF ERYSIPELAS.—In conclusion, I will endeavor to give a comprehensive resume of the treatment of erysipelas as I have presented it.

In the treatment of erysipelas, in all its forms, we have to consider:

First. The constitutional condition which may have predisposed to the disease.

Second. The lesion which has acted as its exciting cause, if the inflammation be traumatic.

Third. The tendency to local destruction of tissue, and the formation and burrowing of pus.

Fourth. The tendency to the typhoid condition, proportionate to the severity of the disease.

Fifth. The danger of septic absorption and toxæmia.

Sixth. The danger of propagation by contagion.

Seventh. The liability to serious deformity from cicatricial contraction.

1. The first indication is to be met by measures tending to correct the cachexia present, if possible. 2. The second, by careful attention to the wound, and rigid antisepsis. 3. The tendency to local destruction of tissue is to be combatted by free incisions adequate to relieve all tension, and the application of hot dressings to maintain the vitality of the part and promote absorption of inflammatory exudate, and also to relieve the pain. The possibility of pocketing and burrowing of pus should be prevented by immediate incision into all purulent collections. 4. The supervention of typhoid symptoms should be prevented, if possible, by proper analeptic and tonic measures, and the liberal use of stimulants. 5. The fifth indication calls for perfect cleanliness and prompt removal of dead tissues which might excite decomposition of the discharges and consequent septic absorption; with the adoption of antiseptic dressing, 6. Transmission of the disease should be prevented by strict isolation of the affected, cleanliness, both in regard to the seat of disease and the instruments used, and on the part of the attendants, with the conscientious use of antiseptics. 7. Deformity may be prevented by careful management of the ulcers resulting from the disease, and the judicious use of skin-grafting. Various mechanical contrivances may be necessary in certain situations to prevent contraction.—*G. Frank Lydston, M.D., in Chicago Med. Jour. and Ex.*

---

Surgery of the Arteries.—1. Bleeding from an accessible artery should be checked by twisting or tying both ends of the vessel.

2. Moderate hemorrhage from one not readily reached, should be controlled by rest, position, local pressure, compression, etc.

3. Severe hemorrhage from an artery not controllable by pressure, etc., requires that the vessel be sought in the wound and both ends tied or twisted.

(*a*) An important exception is to be noted in some cases of wounds of the hands or feet: the danger of damaging important structures may lead the surgeon to tie or twist the brachial or femoral rather than seek the wounded branches of the palmar of plantar arches.

4. In fractures, complicated by wound or rupture of a large artery, the surgeon should at once cut into the swelling, turn out the clots, twist or ligate the central and peripheral ends of the arterial trunk.

5. In pulsating tumors, following wounds or injuries of arteries, should rest, position, local pressure or general compression fail to check their growth or diminish their size, an operation should be performed, the tumor opened, clots removed and the vessel tied or twisted above and below the lesion.

6. When a wound or injury of an artery has led to extravasation, no operation should be performed, so long as rest, position, pressure or compression can restrain further hemorrhage. (*a*) An exception to this rule is to be noted in cases where the amount of blood in the tissues is so great as to threaten their integrity by gangrene, or where, after checking further increase, the above measures fail to cause a diminution in the size of the tumor, despite the length of time they have been employed.

7. If the extravasation caused by arterial wound or injury cannot be held in check and further hemorrhage prevented by position, rest, local pressure or general compression, the tumor should at once be cut open, the clots removed, and the vessel tied or twisted above and below the point of injury.—*Reuben A. Vance, M.D., in Columbus Med. Journal.*

---

Death by Chloroform.—The chloroform was given upon a towel folded funnel-fashion. The towel was at first held a little distance from the face, until the patient grew accustomed to the vapor and was habituated to the proper inhalation The usual period of excitement came on, with some struggling of the arms and rolling of the body. One of the female attendants helped to control these movements, and in a short time relaxation began to be evident, with the slightest stertor of breathing. Less than three drachms of chloroform had been used. I at once suspended the chloroform, passed the towel over to the nurse, who was at the bedside, and a little removed, and asked her to hold it where she was. I felt no apprehension about the patient, and moved to the door separating the chamber from the parlor, and called to Drs. Simons and Pelzer, my assistants, to enter. I now took my position at the foot of the table, while my assistants remained at the side, and began to put the patient into the semi-prone and lateral position for operation. I little thought that during the few seconds of absence the cumulative effects of the drug would be exhibited. Glancing at the face of the patient, I suddenly discovered that it was cyanosed, and the eyes staring and fixed. I called to Dr. Simons to notice if the breathing was right, and almost simultaneously we advanced to the patient's head. I saw that the respiration was embarrassed, and heard a gurgling noise coming from the presence of mucous secretions in the bronchii. Dr. Simons raised the head of the patient, and turned the body partly over into the supine position. I threw up the windows, dashed cold water upon the face and chest, slapped the surface smartly, depressed the head, while the body and lower extremities were raised, injected brandy, and subsequently liquor ammonia and brandy, subcutaneously. Towels were wrung out of very hot water and applied over the cardiac region. Used galvanic battery as soon as this could be secured. Finally, noticing that the respiratory movements were now entirely arrested, also the action of the heart, while the veins of the neck were greatly distended, I opened, first, a vein at the bend of the arm, and afterwards the right external jugular, hoping that, by removing some of the dark blood from the cavities of the heart, this organ would have a better chance for contraction. All to no purpose—the heart remained paralyzed, and we had soon to realize the fearful fact that death had supervened—*R A. Kinlock, M.D., in Philadelphia Med. News.*

The "cone" is not a safe method of administering chloroform, and should never be employed.

---

OIL OF TURPENTINE IN SMALL DOSES.—The *Journal de Therapeutique*, of Paris, publishes an interesting review of an essay upon the therapeutic properties of oil of turpentine in small doses, by Dr. Ingigliardi.

Taken in small doses frequently repeated, oil of turpentine, according to this observer, causes slight cerebral disturbance, and is followed by decidedly favorable results, especially in cases of collapse, the coma of suntroke, the adynamic condition of cholera, and of infectious fevers.

It affects markedly the spinal cord, the medulla oblongata and the motor nerves, and excites energetic contractions in certain muscular organs, such as the heart and the uterus. The calibre of the blood vessels is diminished by its action, and its hemostatic influence in pulmonary as well as vesical and uterine hemorrhage is thus explained.

It is a very valuable means, also, of controlling reflex action in certain neuroses, like asthma, tetanus and epilepsy; it is possible, too, that it would be of service in the transitory mania of alcoholism.

On account of its effect upon the brain, it is an inestimable palliative in the agonising pains of rheumatic neuralgia, sciatica, tic douloureux, and iodopathic visceral forms of neuralgia. Its sedative power, moreover, in the cephalalgia of anæmic women is pointed out and its favorable action attributed to stimulating the circulation of blood in the brain.

Oil of turpentine acts, again, upon the vaso-motor nerves, and consequently upon the circulatory system, with special tonic influence upon the mucous membranes, diminishing and modifying the catarrhal secretions of the respiratory, digestive and urinary tracts.

It can be used with advantage in dropsies where there is no inflammation of the kidneys present, because it provokes this organ to greater activity and relieves its torpid condition. In like manner, every variety of œdema has been benefitted by it, as well as hydrothorax and ascites.

Oil of turpentine, in fine, possesses, according to this author, specific anti-spasmodic, anti-catarrhal, hemostatic and antiseptic properties. It should be administered in weak doses, not exceeding 50 centigrammes to 1 gramme a day, either before or immediately after meals.—*Therapeutic Gazette.*

---

Intussusception.—The child vomited as the pains appeared, and the matter thrown from the stomach was becoming very offensive. At the intervals between the pains he slept continuously a deep heavy sleep. Sordes had collected upon the teeth, the breath was offensive and the face bore a distressing pinched appearance, in fact, all the evidences of coma and approaching death were at hand. I explained to the people what I considered to be the nature of the case, and expressed grave doubts as to any hope of recovery.

As Dr. D. T. Nelson was to meet me in some clinical work at that time I invited him to see the child and he concurred in the opinion I had expressed and advised laparotomy, suggesting that a skillful surgeon be called, whereupon I called Dr. Christian Fuger to see the case with me. I had concluded to make an effort to relieve the child by warm water injections, but thought I would await the conclusions of Dr. Fuger. He came late in the evening and by his examination placed the diagnosis beyond doubt and agreed to perform abdominal section in the morning, but advised that I try my original plan in the meantime; consequently after his departure I placed the child in the dorsal position, elevated the hips, giving the body an incline of about 30° and with a Davidson syringe commenced the injection of tepid water, at the same time following the course of the intestine with my other hand upon the abdomen. I could distinctly recognize the firm obstruction in the large intestine with the tympanitic distension above and the gradually expanding walls below under the force of the injection.

This was kept up until some eighteen or twenty ounces of water had been used when the constriction gave way and with a rush of water and gases the distension subsided. A few minutes later the bowels moved and then the child went to sleep and rested until morning. The recovery was very slow because the slightest indiscretion in diet would start a diarrhœa which often became quite annoying, but the recovery was complete with no further trouble.—*E. P. Murdock, M.D., in Western Med. Reporter.*

---

Foreign Bodies in the Air Passages.—1. When a foreign body is lodged either in the larynx, trachea, or bronchia, the use of emetics, errhines, or similar means should not be employed, as they increase the sufferings of the patient and do not increase his chances of recovery.

2. Inversion of the body and succussion are dangerous, and should not be practiced unless the windpipe has been previously opened.

3. The presence simply of a foreign body in the larynx, trachea, or bronchia does not make bronchotomy necessary.

4. While a foreign body causes no dangerous symptoms, bronchotomy should not be performed.

5. While a foreign body remains fixed in the trachea or bronchia, as a general rule bronchotomy should not be practiced.

6. When symptoms of suffocation are present or occur at frequent intervals, bronchotomy should be resorted to without delay.

7. When the foreign body is lodged in the larynx, there being no paroxysms of strangulation, but an increasing difficulty of respiration from edema or inflammation, bronchotomy is demanded.

8. When the foreign body is movable in the trachea, and excites frequent attacks of strangulation, bronchotomy should be performed.—*J. R. Weist, M.D., in American Practitioner.*

---

Otology for General Practitioners.—Before closing, I will again tabulate the diseases which the general practitioner is most liable to meet.

*The External Ear.*—1st. Collections of wax.

2d. Circumscribed inflammation (boils).

3d. Diffuse inflammation.

4th. Foreign bodies.

5th. Aspergillus.

*Middle Ear.*—1st. Acute aud subacute catarrh—*non suppurative.*

2d. Chronic catarrh—*non suppurative.*

3d. Acute catarrh—*suppurative.*

4th. Chronic catarrh—*suppurative.*

5th. Mastoid disease.

6th. Polypi.

*Internal Ear.*—Syphilitic disease principally.

Bearing these points in mind, I feel perfectly safe in saying that any fair practitioner, after a little preliminary work with the head-mirror and speculum, can make as accurate a diagnosis as there is ever a necessity for, and can treat successfully many cases of disease that are at present neglected or misunderstood.—*F. Tipton, M.D., in Virginia Med. Monthly.*

---

The Blue Color of Water.—The *Photographic News* states that Mr. Aitken has been studying the blue color so characteristic of the Mediterranean and the Lake of Geneva, and his conclusions are embodied in a paper presented last month to the Royal Society. Mr. Aitken begins by saying that two solutions have been offered of this puzzling problem—the one explained the color as due to reflection of small suspended particles which did not reflect the lower rays of the spectum, and the other that the color was the result of the absorbent action of the water itself upon the white light, before and after reflection of these particles. The latter theory Mr. Aitken holds to be the true one. The smaller the number of white reflecting particles the darker and greener the water appears to be. Mr. Aitken having been successful in turning the still green water of Lake Como into a bright blue by scattering finely divided chalk in the middle of the lake.—*Independent Practitioner.*

---

Diagnosis of Vesical Calculus.—Volkmann's investigations (*Centralblatt fur Chirurge*) on the diagnosis of stone in the bladder in children is of importance to the general practitioner as well as to the surgeon especially interested in the treatment of these cases. He states that the rectal bimanual examination for stone is of great value. The patient is thoroughly placed under the influence of an anæsthetic until the abdominal muscles are completely relaxed. The bladder is to be empty or but slightly distended; two fingers of the left hand are to be carried as high up into the rectum as possible; the right hand presses upon the abdomen above the symphysis pubis and forces the bladder down towards the rectum until both hands meet. In this way even small calculi can be readily detected, but it needs considerable practice and experience to determine the size of the stone, Volkmann finding at first that the stone turned out to be larger on extraction than he had expected on examination, although distinctly feeling it between his fingers. The best way to determine the size is to lift it up on to the os pubis and hold it there; this Volkmann has done in his last four cases, the last stone which he palpated in this manner being as large as a chestnut.—*Boston Med. and Surg. Journal.*

---

The Pathology and Treatment of Epidemic Cerebro-Spinal Meningitis.—Dr. Karl Jaffe makes to the *Deutsches Archiv fur Klin. Med.* an interesting contribution to the knowledge of this subject. He regards epidemic meningitis as an infectious disease characterized by an altogether specific virus, appearing sporadically as well as epidemically, and extended just as well through miasatic influences as by contagion. The *origin* of the *materies morbi* is as yet undiscovered. As regards the season of the year when most prevalent, it is generally believed that most of the cases occur in the winter and spring; but of the cases reported by the author seven were in the spring, eight in the summer, and only two in the winter. As to sex, the usual preponderance of males is noted, thirteen males to four females; and as to the age, more than half the cases occurred between the twentieth and thirtieth year. Nothing definite was ascertained with reference to the relations of the residence and mode of life of the pa tients; at least, no useful conclusions could be drawn from the investigation, and the writer was obliged to fall back upon the nature of the virus itself as the only explanation possible. That such exists can be considered as demonstrated; it also has lately become highly probable that it is less of a miasm than a *contagion*; but whether this contagion is fixed or volatile, living or not, or entirely parasitic in the modern sense, is a question of the highest interest, but whose solution is probably at present only in its first beginning. Careful examinations of the blood, and of the exudation into the pia, after the method of Koch and others, were made, but with only a negative result. Unless further observation yields more positive results, the conclusion is warranted that epidemic meningitis is not a parasitic infectious disease. That it has many points of similarity with such diseases the author admits· Indeed, he does not deny that in some forms a true pyæmic character is seen; twice he observed endocarditis, five times joint-affections, and once muscular abscess; he concludes, however, that the literature of the last few years has taught us to be cautious in regard to pathological conclusions based upon analogies. He believes that science will be rendered better service by an open and honest "Ignoramus" than by reflections and speculations that busy themselves in setting in place of anything unknown something just as little proved.

The *diagnosis* of sporadic cases presents difficulties, especially in the first few days. The differential diagnosis requires the following to be taken into consideration:

idiopathic (traumatic) spinal or cerebro-spinal meningitis, tubercular basilar meningitis, typhoid fever, intermittent, asthenic pneumonia, tetanus, delirium tremens, and acute mania.

From traumatic meningitis the specific form may be distinguished by the history; but it must not be forgotten that it is precisely the wounded that are especially liable to suffer from this infection: on the other hand (as one of the cases herein reported witnesses), an individual suffering with cerebro-spinal fever may receive accidental injuries to the skull and yet die of the original disease.

From tubercular meningitis the epidemic form cannot often be distinguished: two cases are mentioned which were mistaken for the latter, and it was only after an autopsy that their true nature was detected.

From typhoid fever meningitis may very soon be distinguished by the want of gastric symptoms, the absence of enlarged spleen, and the spinal symptoms, which in typhoid never appear in such intensity.

An intermittent may be soon recognized through the effects of quinia. Severe pneumonias, especially when they appear during an epidemic of meningitis, may be mistaken for the latter; furthermore, the possibility of a combination of pneumonia with meningitis as a complication must be borne in mind. Only the further course of the disease can clear up this point.

The same observation holds good for tetanus, which, moreover, can only very seldom be brought in question.

For delirium tremens the points of distinction have already been stated above.

Finally, as regards the psychoses (acute and transitory mania, epileptic insanity, and acute delirium) occurring during an epidemic, it is only necessary to bear in mind the possibility of their occurrence: careful and sufficient observation will enable us just here to avoid diagnostic errors the soonest.

The *therapeutics* of this disease are quickly summed up. The author approves of the advice of the older observers with regard to the ice-treatment applied to the spine, and the use of narcotics, without which we can scarcely succeed even in the lightest cases. Blood letting has only a very transitory effect. Of remedies, laxatives, especially calomel in large doses, are especially valuable. Mercurial inunctions in one case were without any influence worth mentioning. Prolonged bathing cannot generally be carried out; yet tepid and cool baths, when the temperature is high, are very useful. The medicamental antipyretics (quinia, salicylic acid, and also sodium benzoate) may be entirely discarded, because they are more uncertain than the baths, and they ruin the already weakened digestion. He found that the sick were always worse after their use, in the cases in which they were tried, so he finally gave it up. This medical nihilism will appear justified if we consider how largely those affected with meningitis perish, especially with the rapid form of the disease, how protracted is the convalescence, and what lasting injury must follow the use of every remedy and every means (we might here also include blood-letting) which in any way alters the digestive tract and affects the tissue changes of the patient.—*Philadelphia Med. Times.*

---

THE CONTAGIOUSNESS OF PHTHISIS.—With regard to the popular opinion of the contagiousness of phthisis, which has prevailed for a long time, more or less, in certain countries, and especially in Italy, it may be said that it probably has its foundation in the fact that in most parts of the world more people die from phthisis than any other single disease, and the number of accidental coincidences would naturally lead to an occasional conviction that the disease had been conveyed by contagion; but on the other hand, the immense number of cases in which personal exposure leads to no such results has always been to most medical men a sufficient evidence that it is not contagious.

Physicians ought, we think, to be exceedingly careful how they speak to the public of matters of so great importance. It is no light matter to declare to a mother that her tuberculous child must be separated, not temporarily, as in small-pox, but permanently, from herself and family to ensure their safety. That for the same reason the husband and wife should be separated, as might properly be done in a case of malignant diphtheria. Nevertheless, if the doctrine of the contagiousness in regard to phthisis is established, the Michigan Board of Health is right in saying that "many of these rules," made to prevent the spread of small pox, should always be applied to these cases; but we affirm that it is not yet proven, nor indeed, in our opinion, rendered probable.—*Frank H. Hamilton, M. D., in Med. Gazette.*

---

How to Cook Rice.—Rice is becoming a much more popular article of food than heretofore. It is frequently substituted for potatoes at the chief meal of the day, being more nutritious and much more readily digested. At its present cost, it is relateivly cheaper than potatoes, oatmeal or grain grits of any kind. In preparing it only just enough cold water should be poured on to prevent the rice from burning at the bottom of the pot, which should have a close-fitting cover, and with a moderate fire the rice is steamed rather than boiled until it is nearly done; then the cover is taken off, the surplus steam and moisture allowed to escape, and the rice turns out a mass of snow-white kernels, each separate from the other, and as much superior to the usual soggy mass, as a fine mealy potato is superior to the water soaked article.—*Med. and Surg. Reporter.*

---

The Sequelæ of Measles.—In a recent number of the *Medical Record* Dr. V. P. Gibney, of New York, discussed the sequelæ of measles, with special reference to the development of a strumous diathesis. He draws the following conclusions: 1. Measles is not by any means a trival disease. 2. Measles, and indeed any of the exanthemata, with hooping-cough especially included, are to be dreaded in patients suffering from chronic bone, and joint-diseases commonly known as scrofulous. 3. Measles and hooping-cough take precedence among all diseases of infancy and childhood in the evolution of a hereditary strumous diathesis. 4. A strumous diathesis may be *caused* by an attack of measles or of hooping-cough in a child whose family history, both paternal and maternal, is absolutely free from hereditary diseases.—*J. B. M., in Louisville Med. News.*

